# Psychological struggles in tunisian Infertile couples: A gender Perspective

**DOI:** 10.1192/j.eurpsy.2024.351

**Published:** 2024-08-27

**Authors:** K. Mahfoudh, F. Askri, S. Hamzaoui, A. Ouertani, U. Ouali, A. Aissa, R. Jomli

**Affiliations:** Psychiatry “A”, Razi Hospital, Manouba, Tunisia

## Abstract

**Introduction:**

Defined by the World Health Organization (WHO) as the inability to conceive after a year of unprotected sexual intercourse, infertility remains a current and compelling topic of interest for both scientists and the general public.

Over the past few decades, the prevalence of infertility, regardless of its cause, has significantly increased. Furthermore, it affects approximatively 15% of tunisian couples. However, previous studies have primarily assessed the psychological impact on women, leaving a gap in understanding gender differences.

**Objectives:**

Our study aims to compare the psychological impact of infertility between genders in a Tunisian sample.

**Methods:**

We conducted a cross-sectional study in a public hospital specializing in Assisted Reproductive Technology (ART) from August 30th to December 1st, 2022, involving sexually active infertile couples who had been under observation for at least one year. The participants provided information related to socio-demographic data. Additionally, we used the Hospital Anxiety Depression Scale (HADS) to assess anxiety and depression, and the Fertility Quality of Life (FertiQol) questionnaire to evaluate the quality of life. These questionnaires were administered in the Tunisian dialect.

**Results:**

A sample of 60 infertile couples were recruited to this study. Primary infertility was present in 97% of cases and male infertility was the most common cause, accounting for 35%. Our findings revealed that women experienced higher rates of depression (35%) and anxiety (52%) compared to men (15% and 28%), with a statistically significant difference (p ≤ 0.001).

Furthermore, women reported a significantly compromised overall quality of life, particularly in the context of treatment-related aspects (p=0.03).

Notably, anxiety was identified as a significant risk factor for reduced quality of life among women (B = -5.27). In contrast, lower socioeconomic status was associated with diminished overall quality of life in men (B = -7.09).

**Conclusions:**

It is important to consider gender differences in the management of infertility in order to guide and target psychological interventions and to improve the quality of life of infertile couples.

**Disclosure of Interest:**

None Declared

